# A Rare Case of Intraosseous Fibrolipoma of the Mandible: Diagnosis and Treatment

**DOI:** 10.1155/2015/519824

**Published:** 2015-04-05

**Authors:** Andrea Castellani, Gabriele Bocchialini, Luca Ferrari

**Affiliations:** Department of Maxillo-Facial Surgery, Civil Hospital, 25123 Brescia, Italy

## Abstract

Lipomas are common soft tissue tumors. Intraosseous lipoma is a rare lesion that constitutes not more than 0.1% of bone tumors. It can occur anywhere in the body and there have only been a few cases found in the mandible. Intraosseous fibrolipoma of the jaw is an uncommon histological variant of the classic lipoma and to the best of our knowledge only one case has previously been reported in the literature. The cause of this lesion is uncertain. Clinically the lesion is generally asymptomatic and its radiographic feature is a well-defined radiolucency. Surgery is the treatment of choice. We present a case of an intraosseous fibrolipoma in the right mandibular ramus in a 25-year-old female.

## 1. Introduction

Lipoma is a benign mesenchymal soft tissue tumor of mature adipose tissue with no evidence of cellular atypia.

Lipomas are uncommon in the oral cavity and the incidence of this neoplasm is between 1% and 4.5% of all benign lesions [1].

Based on the literature, the overall incidence of intraosseous lipoma represents less than 0.1% of all bone tumors [2–4] and only 20 cases, including this one [5–7], have been reported in the mandible since 1948 [8].

Intraosseous lipoma is an uncommon disease. Roux [9] described the first oral lipoma in 1848 and Rose II illustrated a fibrolipoma of the jaw and neck in the 1906 [10]. Then, in 1948, Oringer [8] showed the first report of an intraosseous lipoma of the mandible and in 1957 Newman [11] made the first detailed scientific description of an intraosseous fibrolipoma of the mandible.

Fibrolipoma has been rarely described and, according to the WHO classification [12], is a variant of conventional lipoma [13]; it is characterized by mature adipose tissue interspersed by bands of connective tissue [14].

According to the latest review done by de Freitas Silva et al. in 2011 [5] (and to the best of the authors' knowledge) there is only one case in the literature of intraosseous fibrolipoma of the mandible [11].

Stating the importance of each new case of intraosseous lipoma of the mandible, we report a new case of fibrolipoma affecting the right mandibular ramus.

## 2. Case Report

A 25-year-old female was referred to the Unit of Maxillo-Facial Surgery at the Civil Hospital in Brescia by her dentist for an evaluation of a painless radiolucency in the right mandibular ramus.

The extra- and intraoral evaluation reveal no swelling, cortical expansion, or mucosal anomalies; there was no limitation of mouth opening, no alteration of the mandibular movement, and no history of trauma or cancer in her family. No damage of the inferior alveolar nerve was revealed.

**Figure 1:**
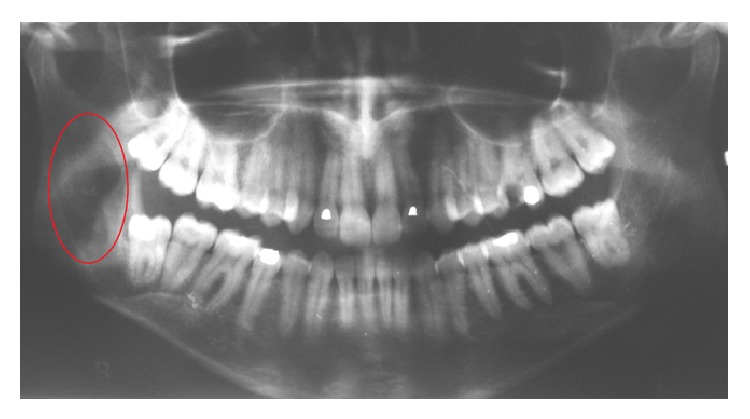
OPT show radiolucency in the right mandibular ramus.

The orthopantomography showed a radiolucency in the right mandibular ramus (Figure 1).

**Figure 2:**
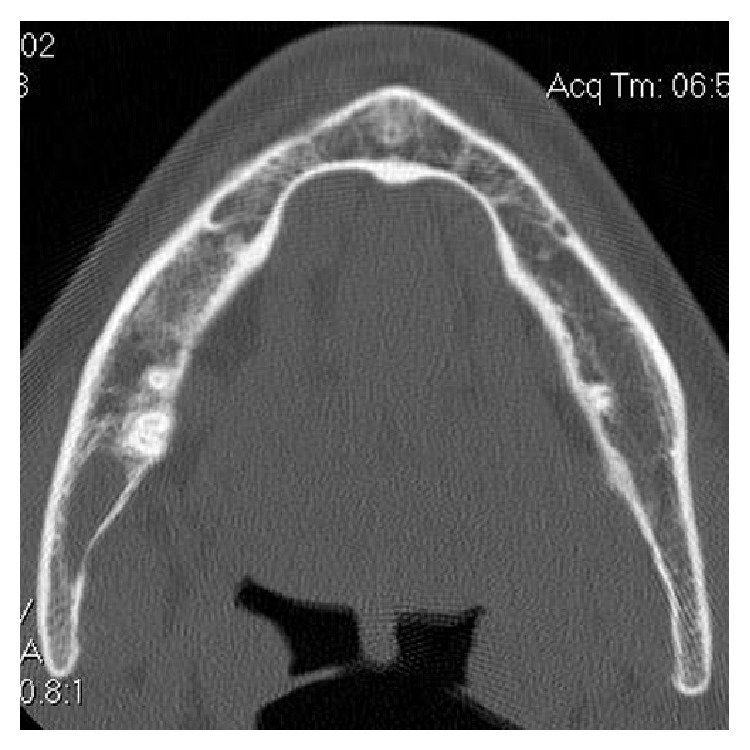
Tc axial scan revealed a well circumscribed unilocular image involving the mandibular ramus.

A computer tomographic (CT) scan revealed a well circumscribed radiolucent unilocular image with sclerotic margins involving the mandibular ramus (Figure 2).

**Figure 3:**
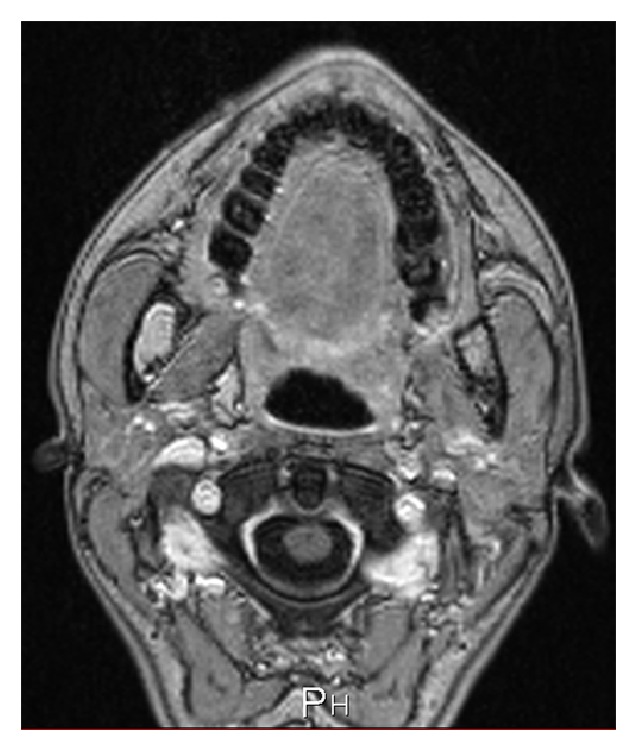
MRI showed the lesion with signal reduction in the sequences with fat suppression.

**Figure 4:**
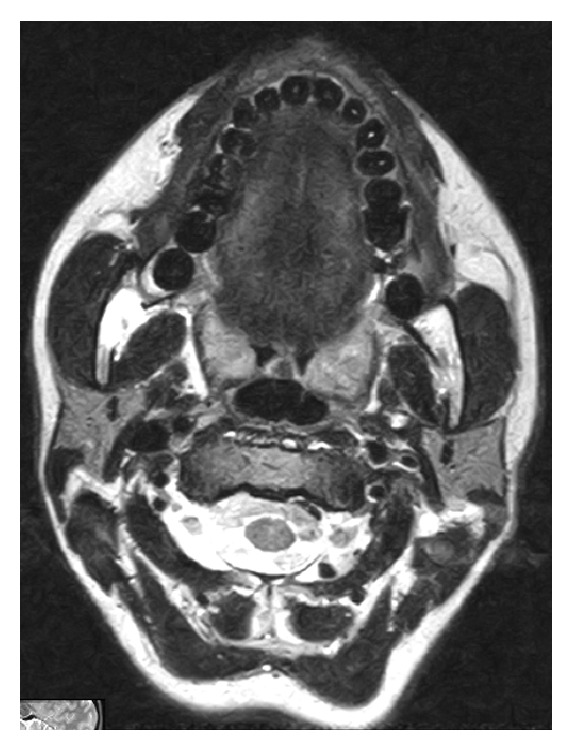
MRI showed the lesion with signal reduction in the sequences with fat suppression.

Magnetic resonance imaging (MRI) showed a hyperintense neoformation in the T1 and T2 sequences; in the sequences with fat suppression the lesion showed a signal reduction (Figures 3 and 4).

As the CT scan and MRI strongly indicated a benign lesion, no exploratory biopsy was performed.

**Figure 5:**
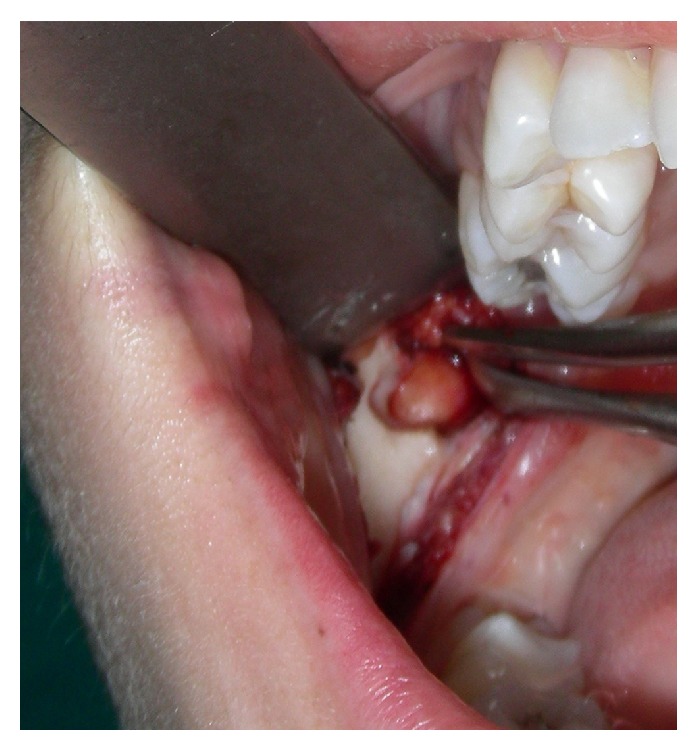
Intraoperative view.

Under local anesthesia (Mepivacaine 0.2% with 1 : 100.000 adrenaline), an intraoral approach was done. Then an incision along the right external oblique line was performed, the periosteum was elevated, and the bone was drilled until the lesion was exposed; therefore the lesion was removed after blunt dissection (Figure 5).

The perilesional curettage after the total removal of the tumor was performed.

During this procedure, trauma to the inferior alveolar nerve and lingual nerve was avoided.

**Figure 6:**
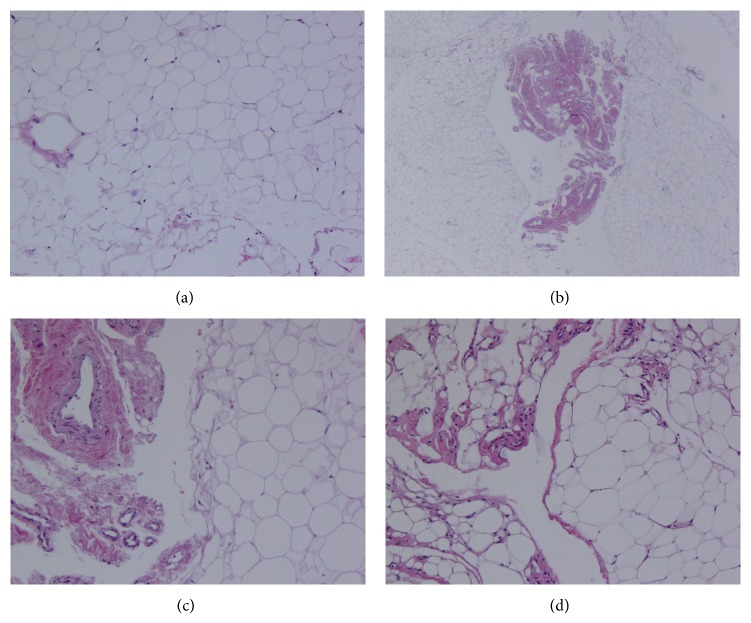
Histopathological view.

The histopathological diagnosis of the lesion was fibrolipoma (Figure 6).

There was no evidence of recurrence at a 1-year follow-up.

## 3. Discussion

In agreement with Cakarer et al. (2009), intraosseous lipomas may arise from the soft tissue adjacent to the bone or may occur in an intramedullary location [15].

The aetiology is not clearly understood; theories such as trauma, infarction, inflammation, and nutritional problems have been proposed [3, 15–17]. It is usually accepted that it is a true benign tumor of the medullary adipose tissue [2].

Histologically, fibrolipoma is a variant of lipoma, such as spindle cell lipoma, intramuscular or infiltrating lipoma, angiolipoma, sialolipoma, pleomorphic lipoma, and myxoid and atypical lipomas [18, 19].

Intraosseous lipomas were classified and subdivided by Milgram [4] into three groups, depending on the degree of involution.

**Stage 3 I.**

Stage I is tumors of viable fat cells.

**Stage 3 II.**

Stage II is transitional cases composed partly of viable fat cells but also demonstrating fat necrosis and calcification.

**Stage 3 III.**

Stage III is lesions demonstrating necrotic fat, calcification of necrotic fat, variable degrees of cyst formation, and reactive woven bone formation.

In contrast to the other cases present in literature described by Newman [11], our patient is a 25-year-old female without a history of trauma and the lesion site was in the right ramus of the mandible. In common with Newman [11], the patient was asymptomatic and the orthopantomography showed a well-defined radiolucency.

The lesion was found accidentally during routine radiographs. Hypoesthesia, pain, and swelling can be associated with these lesions and depend on the location and on the size of the tumor.

A radiograph of intraosseous lipoma is usually uncharacteristic and presents a cystic lesion with and increased radiolucency surrounded by a sclerotic rim [2, 15, 17]; computer tomography and magnetic resonance imaging allow a more precise evaluation of the morphology of the lesion [20–22].

In our case MRI showed a hyperintense neoformation like fat tissue in the T1 and T2 sequences; in the sequences with fat suppression the lesion showed signal reduction. For these reasons and for the intraoperative macroscopical aspect of the lesion, the total excision of the neoformation was performed.

The differential diagnosis includes keratocystic odontogenic tumor, liposarcoma, simple bone cyst, bone marrow defect, early benign fibroosseous lesion, central giant cell granuloma, calcifying epithelial odontogenic tumor, odontogenic myxoma, cartilaginous tumor, and ameloblastoma [2].

## 4. Conclusion

Surgery has been proposed as the therapy of choice [15].

No recurrence or malignant changes of intraosseous lipomas in the maxillofacial region have been reported in the literature [15].

As intraosseous lipomas of the mandible are rare, the diagnosis is very important and it is essential that each new case is documented, especially for the fibrolipoma which has an increased growth potential compared to the classic lipoma [14].
